# A systems biology approach to investigating the influence of exercise and fitness on the composition of leukocytes in peripheral blood

**DOI:** 10.1186/s40425-017-0231-8

**Published:** 2017-04-18

**Authors:** Michael P. Gustafson, Ara Celi DiCostanzo, Courtney M. Wheatley, Chul-Ho Kim, Svetlana Bornschlegl, Dennis A. Gastineau, Bruce D. Johnson, Allan B. Dietz

**Affiliations:** 1 Human Cellular Therapy Laboratory, Department of Laboratory Medicine and Pathology, Division of Transfusion Medicine, Mayo Clinic, Rochester, MN USA; 2 Department of Cardiovascular Diseases, Mayo Clinic, Rochester, MN USA; 3 Department of Immunology, Mayo Clinic, Rochester, MN USA; 4 Laboratory Medicine and Pathology, Mayo Clinic, Hilton 2-74B, Rochester, MN 55905 USA

**Keywords:** Exercise immunology, Fitness, Peripheral blood leukocytes, T cells, NK cells, Monocytes

## Abstract

**Background:**

Exercise immunology has become a growing field in the past 20 years, with an emphasis on understanding how different forms of exercise affect immune function. Mechanistic studies are beginning to shed light on how exercise may impair the development of cancer or be used to augment cancer treatment. The beneficial effects of exercise on the immune system may be exploited to improve patient responses to cancer immunotherapy.

**Methods:**

We investigated the effects of acute exercise on the composition of peripheral blood leukocytes over time in a male population of varying fitness. Subjects performed a brief maximal intensity cycling regimen and a longer less intense cycling regimen at separate visits. Leukocytes were measured by multi-parameter flow cytometry of more than 50 immunophenotypes for each collection sample.

**Results:**

We found a differential induction of leukocytosis dependent on exercise intensity and duration. Cytotoxic natural killer cells demonstrated the greatest increase (average of 5.6 fold) immediately post-maximal exercise whereas CD15^+^ granulocytes demonstrated the largest increase at 3 h post-maximal exercise (1.6 fold). The longer, less intense endurance exercise resulted in an attenuated leukocytosis. Induction of leukocytosis did not differ in our limited study of active (*n* = 10) and sedentary (*n* = 5) subjects to exercise although we found that in baseline samples, sedentary individuals had elevated percentages of CD45RO^+^ memory CD4^+^ T cells and elevated proportions of CD4^+^ T cells expressing the negative immune regulator programmed death-1 (PD-1). Finally, we identified several leukocytes whose presence correlated with obesity related fitness parameters.

**Conclusions:**

Our data suggests that leukocytes subsets are differentially mobilized into the peripheral blood and dependent on the intensity and duration of exercise. Pre-existing compositional differences of leukocytes were associated with various fitness parameters.

**Electronic supplementary material:**

The online version of this article (doi:10.1186/s40425-017-0231-8) contains supplementary material, which is available to authorized users.

## Background

In the field of exercise immunology, the “inverted J hypothesis” has been used to describe the effects of exercise on the function of the immune system whereby regular moderate exercise results in improved immune function and decreased risks of disease susceptibility [1]. Conversely, extensive overtraining may lead to immune suppression and increased risks of disease susceptibility. Data from epidemiological studies suggest that there is a correlation between a moderate level of physical activity with lower levels of bacterial and viral infections as well as decreased cancer incidence and mortality [2, 3]. Nearly ¼ of cancer cases worldwide are thought to be the result of excess weight, sedentary lifestyle, and inactivity [4]. These factors stress the immune system by overproduction of sex hormones, excess inflammation and hormones, and decreased overall immune function. Although higher levels of physical activity support the immune system, lead to better prognosis in cancer, and lower overall incidence of cancer and infectious disease, it is not clear how long-term exercise results in improved immune health. Indeed, conflicting reports abound regarding the long term effects of exercise on the immune system [5]. Although many variables likely contribute to this observation, including gender, age, diet, and genetics, the methods to quantify physical activity over time will need to be refined to accurately assess the contributions of exercise to overall immune health.

There are more consistent immune system changes to acute exercise. The rapid and brief accumulation of leukocytes in peripheral blood during and immediately after exercise has been remarkably reproducible (reviewed by Freidenreich and Volek [6]). While natural killer (NK) cells and CD8^+^ cytotoxic T cells appear to be the leukocyte subsets with the largest increases [7], other populations including granulocytes, monocytes and B cells also increase to varying degrees. How these changes lead to both beneficial and harmful responses to the stress of exercise remains under intense investigation. The mechanisms controlling the redistribution of leukocytes are complex and involve the coordination of hemodynamic shear forces, adrenergic stimulation, lymphocyte trafficking through the changes of adhesion molecules, and peripheral blood leukocyte homeostasis [8, 9]. However, several confounding factors contribute to the difficulties in comparing results from one study to another. These include, but are not limited to, the intensity and duration of exercise, standardized methods to measure the output or workload in response to exercise, the phenotypes and functions of the immune cells analyzed, the timing of sample collections, and sample processing and analysis.

Here, we investigated the immune response in terms of the changes of the quantity and composition of peripheral blood leukocytes in healthy adult males after two distinct exercise regimens. The first event was a brief high intensity (maximal) exercise and the second was a longer less intense exercise (endurance). We also investigated whether the physical activity levels would affect the mobilization of leukocytes, and secondarily, to assess if we could detect immunophenotypic changes in baseline samples between active and sedentary individuals. Therefore, we included three groups of individuals; very active, active, and sedentary, and tested them with the same exercise regimens. We were interested in changes of specific subsets in cell counts (cells/μl), how subsets changed in relation to each other, and how the composition of peripheral blood changes as a system. Therefore, we measured immune cell populations by flow cytometry and used 10-color flow protocols that permit that quantification of all major leukocyte subsets simultaneously. Additionally, we were interested in measuring phenotypes associated with immunosuppression including regulatory T cells (Tregs), checkpoint proteins expressed on T cells (CTLA-4^+^ and PD-1^+^) and immunosuppressive CD14^+^HLA-DR^lo/neg^ monocytes. Finally, we tested the samples for correlations between fitness parameters related to obesity and immunophenotypic changes.

## Methods

### Participants

Fifteen healthy males were recruited for this study. Eligibility requirements for participation included that the individuals be nonsmokers, with no known cardiopulmonary or immune disease, and not taking any steroids or immune modulating drugs. The protocol was approved by the Mayo Clinic Institutional Review Board and all participants gave written informed consent. Subject demographics are provided in Table 1.

**Table 1:** Subject demographics (Means ± SD)

Age (years)	31 ± 4
BMI (kg/m^2^)	25.3 ± 3.1
Lean body mass (kg)	58.3 ± 9.8
% body fat	24.2 ± 8.1
Race (% Caucasian)	53
FVC	5.3 ± 1.0
FVC (%predicted)	100.4 ± 10.8
% sedentary (<1 hr physical activity per week)	33

### Experimental design

Prior to the study, subjects were instructed to wear the Body Media device on their upper arm for one week to track their activity level. Sensors in this device use complex algorithms to estimate physical activity levels and durations by measuring motion, number of steps, galvanic skin response, skin temperature, and heat flux. Participants came to the lab on two separate occasions within 1–3 weeks of each other (mean = 11 days; median = 9 days; range 5–25 days). On visit 1, subjects performed an incremental maximal cycling test and visit 2 an endurance cycling protocol of 45 min at 60% of maximal workload. Peripheral blood samples were collected at four time points for flow cytometry analysis of leukocyte subsets prior to exercise, 2–5 min post exercise, 3 h post exercise, and 24 h post exercise.

### Incremental maximal cycling test

To reduce diurnal variation, participants were scheduled for visit one at approximately the same time each morning. Each participant underwent one DEXA scan (Dual-energy X-ray absorptiometry) which was used to calculate lean body mass (LBM) and percent body fat (%BF). A 5 mL blood draw was performed via antecubital venipuncture and collected in K_2_EDTA tubes (Becton Dickinson). Participants were instructed to not fast or maintain any special diet prior to analysis. Participants performed a functional vital capacity; the maneuver was demonstrated to them and they practiced until they achieved three attempts within 150 mL of each other. The Flow Volume Curves (FVC) prediction equation was adapted from Knudson et al. [10]. Next, the subjects performed an incremental cycling test. Gas-exchange, heart rate (HR), blood pressure (BP), 12-lead ECG, and Rate of Perceived Exertion (RPE) were monitored during rest and during each work stage. Performance test began at 50W and increased every 2 min in 30 W stages until exhaustion (when 60–80 rpm could no longer be maintained, their VO_2_ declined, and/or their Respiratory Exchange Ratio was equal to 1.2). This was followed by a 2 min period of recovery cycling and a 5 mL blood draw (post exercise blood draw). Participants were dismissed and asked to return in 3 and 24 h for the additional 5 mL blood draws. Exercise was not permitted until completion of the 3rd post-exercise blood draw.

### Endurance cycling test

Visit 2 was also scheduled similarly to visit 1. After a 2 min warm up on the bike, participants performed a 45 min cycling protocol at ~60% of their maximum workload (determined from visit 1). Gas-exchange, HR, BP, and RPE were monitored intermittently throughout the exercise and 12-lead ECG was monitored continuously. If participant’s heart rate or VO2 drifted too high, or they started getting tired, workload was dropped by ~5–10% to ensure the completion of 45 min of cycling. There was a 2 min period of recovery cycling and then the subjects had 5 mL of blood drawn via antecubital venipuncture (post exercise blood draw). Instructions for the participants were the same as for visit 1 (abstain from exercising until after the 24 h time point and return to the lab for a blood draw at 3 and 24 h).

### Data analysis: incremental maximal cycling test

All physiological data are presented as means ± SD unless otherwise stated. The VO_2_ max represented the average of 30 seconds of the highest VO_2_ values before it began to decrease (usually right before the VO_2_ max test ended). The predicted VO_2_ was used from the Mayo VO_2_ prediction equation = 60–0.5(age) [11]. Lactate was measured before the start of exercise and then at termination of the max test (or within 2 min afterwards) and change was reported as % increase. RER, VE, PET CO_2_, and O_2_ pulse were all averaged over the final minute at maximum workload, and peripheral O_2_ saturation and RPE recorded for the final minute at max.

### Data analysis- endurance cycling test

Gas exchange data was recorded for the first 10 min continuously and the final 2 min of every 10 min segment. Variables over the final minute of each stage (minute 10, 20, 30, and 45) were averaged to provide a value for the entire 45 min work stage for the following variables: VO_2_, HR, Workload, Lactate, RER, VE, PET CO_2_, O_2_ saturation, and RPE. The average O_2_ pulse for the 45 min was calculated by the average VO_2_ divided by the average HR. O_2_ pulse was also calculated for the final minute of exercise (VO_2_/HR) but this value was not significantly different from the average.

### Data analysis- body media

Total energy expenditure (Total EE, average kJ), active energy expenditure (>3.0 MET, average kJ), and average number of steps were collected from Body Media analysis that averages daily values over the period of usage. The fraction of time in each category (sedentary, light, moderate, vigorous, very vigorous) was calculated by averaging the daily amount of time spent in each. The physical activity categories were defined based on METS as follows: Sedentary 0–1.5; Light 1.5-3; Moderate 3–6; Vigorous 6–9; Very Vigorous >9. A MET value of 3 was used as the cutoff between sedentary and active.

### Data analysis- activity level

Questionnaires were used to gather information regarding activity habits including frequency, mode, and duration. The data was quantified by amount of time spent performing each activity each week and by the length of regular exercise. Since there are inherent differences as to how one person trains/exercises and perceives this versus another, there was no method for assessing exercise intensity, and therefore, the reported values were used for qualitative purposes.

### Peripheral blood immunophenotyping by flow cytometry

Peripheral blood samples were processed as soon as possible and stained directly with antibodies without additional manipulations as previously described [12]. Four 10-color antibody flow protocols used for this study were developed by Gustafson et al. [12]. The TBNK/M/G protocol was used with fluorosphere beads to allow the quantitation of phenotypes as cell counts (cells/μl). The other protocols were used (T Cell-1, T Cell-2, and Monocytes-1) to measure specific T cell and monocyte subsets. All antibody information, flow cytometer quality control, instrument settings, and gating strategies are described by Gustafson et al. [12]. The T cell-2 and Monocytes-1 protocols were modified to include other antibodies and are outlined in Additional file 1.

### Statistical analysis

Statistical analyses were performed using Prism software version 5.0 (GraphPad Software). The Repeated Measures Analysis of Variance (ANOVA) tests were used to assess the changes between each of the time points for maximal and endurance regimens. The Bonferroni’s multiple comparison test was used to determine differences between paired time points. The un-paired two-tailed t-test was used and the non-parametric Mann–Whitney tests were used to determine the significance of differences between different groups (active vs. sedentary) where appropriate and the Spearman test was used for correlative data. Principal component analysis was performed using Partek Genomics Suite 6.6 software (Partek Inc. St. Louis, MO). The mean cell count values of 16 phenotypes (CD19^+^, CD56^−^CD16^+^, CD56^+^CD16^+^, CD56^+^CD16^−^, CD3^+^CD56^+^, Lineage^−^, CD4^+^, CD8^+^, CD4^+^CD8^+^, CD4^−^CD8^−^, gamma delta T cells, CD14^+^CD16^−^, CD14^+^CD16^+^, CD14^lo^CD16^+^, CD15^+^CD16^+^, CD15^−^CD16^+^) from 15 individuals were calculated at each time point and normalized to the MAX-Pre values. The ratios were then imported into the Partek software for analysis and analyzed as described elsewhere [13].

## Results

Our study was designed to explore the differences in immune response to acute exercise versus endurance exercise in active and sedentary males. We investigated whether sedentary individuals would respond differently to different bouts of exercise and whether sedentary individuals would display immunophenotypic differences than active individuals. Fifteen males participated in two cycling bouts; a short incremental exercise test to exhaustion on one visit and a 45 min endurance exercise test (cycling at 60% maximum workload) on the second visit.

The subjects were subdivided into three groups based on their maximum aerobic capacity (VO_2_ max), maximum workload during the maximum cycling test (max Watts), their % body fat, and the amount of time they spend active weekly (self-reported sum total of time spent running, cycling, and swimming and/or performing resistance exercise) (Table 2). The participants were ranked among each other within each of these categories and their individual ranks were summed in order to obtain a cumulative rank for each individual. The cumulative ranks ranged from 13 (very active) to 59 (sedentary); 5–24 was considered very active, 25–44 was considered active, and 45–65 was considered sedentary. For the purposes of study recruitment, sedentary individuals were defined as having less than one hour of scheduled physical activity per day (not including walking as part of their daily lives). Responses to the questionnaires and data from the Body Media device are reported in Additional file 2. One of these individuals performed better than expected and thus ranked above two active subjects. However, for the purposes of subsequent data analysis, the subject was still included in the sedentary group.

**Table 2:** Physiological data for incremental maximal cycling test and endurance cycling test

Maximal Cycling Test	Very Active	Active	Sedentary
VO_2_ max (mL/kg/min)	53.3 ± 3.7	44.1 ± 5.2	33.9 ± 10.5
VO_2_ max (% pred.)	120.0 ± 8.9	96.2 ± 11.7	78.3 ± 20.8
Peak workload (W)	302 ± 40	248 ± 54	176 ± 49
Peak HR	172 ± 6	182 ± 11	186 ± 16
Max HR (% pred.)	91 ± 5	95 ± 5	100 ± 9
Lactate (% increase)	785 ± 599	668 ± 385	754 ± 180
RER	1.11 ± 0.07	1.15 ± 0.03	1.20 ± 0.07
VE (L/min)	129.62 ± 25.39	125.73 ± 31.95	96.94 ± 35.44
PET CO_2_ (mmHg)	38.79 ± 2.84	34.34 ± 4.05	36.26 ± 11.36
O_2_ pulse	0.31 ± 0.02	0.24 ± 0.03	0.18 ± 0.05
Peripheral O_2_ Saturation (%)	99 ± 2	98 ± 1	99 ± 1
RPE	19 ± 1	18 ± 2	18 ± 2
Endurance Cycling Test	Very Active	Active	Sedentary
VO_2_ (mL/kg/min)	35.3 ± 1.0	31.6 ± 3.6	23.7 ± 6.6
%of VO_2_ max	67.5 ± 5.3	71.7 ± 3.8	70.5 ± 4.4
HR	136.5 ± 9.5	149.6 ± 14.3	164.6 ± 18.6
Max HR (% pred.)	72.3 ± 6.4	78 ± 7.2	88.6 ± 10.4
Max HR (% of max test Peak HR)	79.5 ± 4.2	82.2 ± 5	88.2 ± 4.2
Workload (W)	179 ± 28	147 ± 33	112 ± 30
% Peak workload	59 ± 2	59 ± 1	56 ± 4
Lactate (% increase)	517 ± 349	438 ± 56	428 ± 231
RER	0.9 ± 0.04	0.92 ± 0.01	0.96 ± 0.02
VE (L/min)	65.31 ± 7.45	63.07 ± 10.92	56.08 ± 10.53
PET CO_2_ (mmHg)	42.17 ± 3.13	39.10 ± 0.46	35.26 ± 5.06
O_2_ Pulse (mL)	0.25 ± 0.03	0.21 ± 0.01	0.14 ± 0.03
Peripheral O_2_ Saturation (%)	99 ± 1	99 ± 1	99 ± 1
RPE	12.4 ± 0.5	13.4 ± 1.7	14.5 ± 2.7

### Physiological data

During the incremental maximal cycling test, all subjects reached an average maximum VO_2_ of 43.5 ± 10.3 mL/kg*min (97.5 ± 21.5% predicted) at a workload of 242 ± 69 W. They reached a maximum heart rate of 179.9 ± 12.8 bpm (95.3 ± 7.2% predicted), a lactate percent increase of 678 ± 319%, and an RPE of 18.3 ± 1.7 during the final minute of exercise indicating that maximal effort was attained. Each participant was then ranked in terms of physical activity and fitness parameters (Table 3).

**Table 3:** Ranking of the physical activity and fitness in the participants

	Subject #	% Body Fat Rank	Peak Watts Rank	Peak VO_2_ Rank	Cumulative Activity Minutes/week	Cumulative Rank
Very active	8	6	1	2	4	13.00
Very active	17	4	2	1	7	14.00
Very active	4	3	3	5	5	16.00
Very active	11	1	7	9	1	18.00
Very active	9	2	6	3	10	21.00
Active	2	7	9	7	2	25.00
Active	1	8	4	6	8	26.00
Active	10	11	5	11	3	30.00
Sedentary	6	5	8	4	15	32.00
Active	16	10	10	8	6	34.00
Active	3	9	11	10	9	39.00
Sedentary	5	12	12	13	15	52.00
Sedentary	12	13	14	12	15	54.00
Sedentary	15	15	13	14	15	57.00
Sedentary	14	14	15	15	15	59.00

### Immune cell population changes in response to exercise

**Fig. 1:**
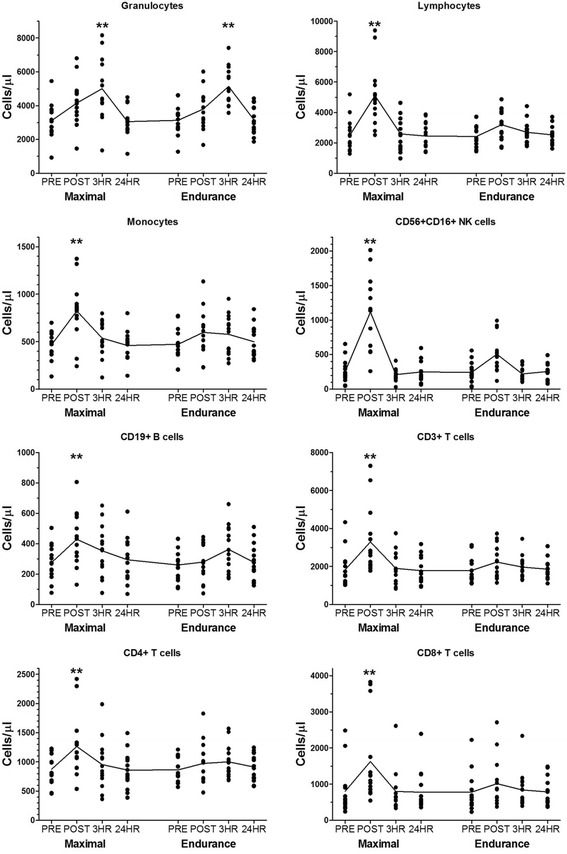
Peripheral blood leukocyte subsets are mobilized distinctly upon two different types of exercise. Blood samples were collected from 15 subjects at the indicated time points after maximal and endurance exercises. Leukocytes were enumerated by flow cytometry. Eight major subsets are shown: granulocytes, lymphocytes, monocytes, Natural Killer (NK) cells, B cells, T cells including CD4^+^ and CD8^+^ subsets. For each subset, the means were significantly different (*p* < 0.0001 by repeated measures ANOVA). ** = *p* <0.01 by the Bonferroni’s multiple comparison post-test of all pairs

Flow cytometry was performed on un-manipulated whole blood at baseline, immediately post exercise, 3 h post exercise, and 24 h post exercise. The 10-color flow protocols allowed us to measure all major leukocyte populations in one tube [12]. Figure 1 shows the cell count values (cells/μl) of the eight major leukocyte populations in peripheral blood in response to maximal and endurance regimens. All of these populations, except for CD15^+^ granulocytes, peaked immediately after exercise and then returned to baseline levels after three hours post-exercise. Granulocytes, however, showed a delayed response by peaking three hours after exercise and returning to baseline levels at 24 h. We observed the largest changes in phenotypes that were measured in cell counts compared to phenotypes measured as a percent of a parent or grandparent population. Of the 24 T cell phenotypes measured as a percentage, 14 were found to have small but significant changes (Additional file 3). Of the 14 phenotypic changes, eight were related to memory T cells phenotypes. Immune-suppressive phenotypes displayed different responses in that Tregs decreased (11.26% of CD4^+^ T cells to 10.18% after maximal exercise; p = 0.006), CTLA-4^+^ cells did not change, and PD-1^+^ T cells increased in both CD4^+^ and CD8^+^ cells (18.28 to 21.43% of CD4^+^ T cells, *p* = 0.0001; and 30.7 to 36.03% of CD8^+^ T cells, p = 0.0001). In monocytes, 6 of 15 phenotypes exhibited small but significant changes with the strongest association related to the increase of CD14^−^CD16^+^ non-classical monocytes (6.77 to 9.26% of CD14^+^ cells; *p* < 0.0001 and Additional file 3). The immunosuppressive CD14^+^HLA-DR^lo/neg^ monocytes increased from 12.58 to 13.72% after exercise (*p* = 0.035). Overall, the leukocyte response to endurance was diminished compared to maximal exercise. Granulocytes were the only cell type that showed significant differences from baseline in the endurance regimen. Similarly these cells peaked at three hours post-exercise and returned to baseline at 24 h. Our data indicates that the extent of leukocyte response to exercise may be more reflective of the change in total numbers of cells that are mobilized rather than percentages of cell population changes within a group.

**Fig. 2:**
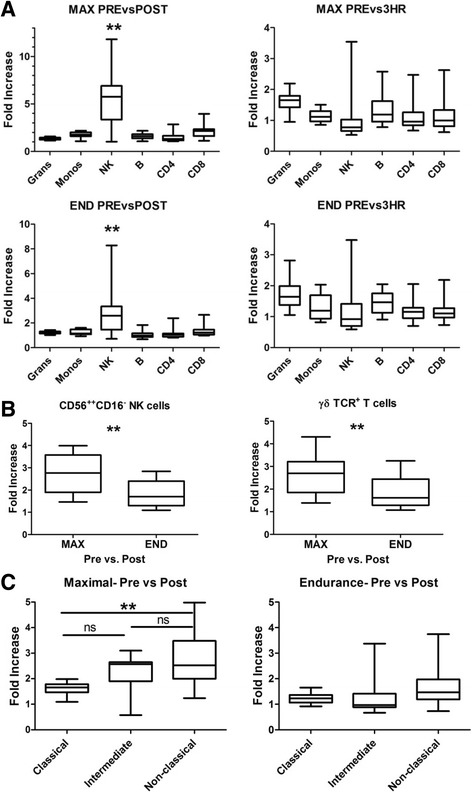
Natural killer cells show the greatest degree of induction upon exercise. **a** The values for each subset at the Post or 3HR time point were divided by the Pre sample value. Box and whisker plots show the 25th and 75th percentiles (box) with median (line in box) and the minimum and maximum values. **b** The changes from Pre to Post for CD56^++^CD16^−^ NK cells and γδ T cells. **c** Changes in the distribution of Classical (CD14^+^CD16^−^), Intermediate (CD14^+^CD16^+^), and Non-classical monocytes (CD14^lo^CD16^+^). The means comparing Pre versus Post were significantly different (*p* < 0.0001 by repeated measures ANOVA). ** = *p* < 0.01 by the Bonferroni’s multiple comparison post-test of all pairs

Having identified the cell types that changed upon exercise, we tested the effects of two types of exercise on the magnitude of induction of leukocytosis for distinct leukocyte subsets. Figure 2a shows the change in six of the eight previously shown immune cell populations (lymphocytes and total CD3^+^ cells not shown) from baseline to post exercise and from baseline to 3 h post exercise. CD56^+^CD16^+^ natural killer cells (NK cells) showed the greatest degree of change versus other cell populations in both the maximal and endurance regimens (Bartlett’s test for equal variances *p* < 0.0001; *p* < 0.01 for NK cell compared to other cell populations). NK cell induction was significantly higher in the maximal test versus the endurance test (5.6 fold increase vs 2.8 respectively; *p* < 0.0001). CD56^br^CD16^−^ NK cells, thought to be precursor cells to CD56^+^CD16^+^ NK cells [14], did not increase as much as CD56^+^CD16^+^ NK cells (2.7 fold in maximal and 1.8 in endurance; p = 0.0017 and p = 0.029 respectively) (Fig. 2b). We looked at the degree of change in other cell types. Another cell type involved in immune surveillance, γδTCR^+^ T cells, was induced after maximal exercise (2.6 fold) (Fig. 2b). Although monocytes were induced to a small degree upon exercise, we investigated whether there was differential induction in three monocyte subsets (Classical- CD14^+^CD16^−^; Intermediate- CD14^+^CD16^+^; Non-classical- CD14^lo^CD16^+^) [15]. Immediately post maximal exercise; non-classical monocytes were induced to the highest degree and were higher than classical monocytes (2.7 fold compared to 1.6; *p* < 0.01) (Fig. 2c). As observed with other cell types, there was no difference in the attenuated induction after the endurance exercise. These data highlight the distinct coordination of the mobilization of immune cells in response to different modes of exercise.

### The peripheral blood leukocyte system responds distinctly to different types of exercise

**Fig. 3:**
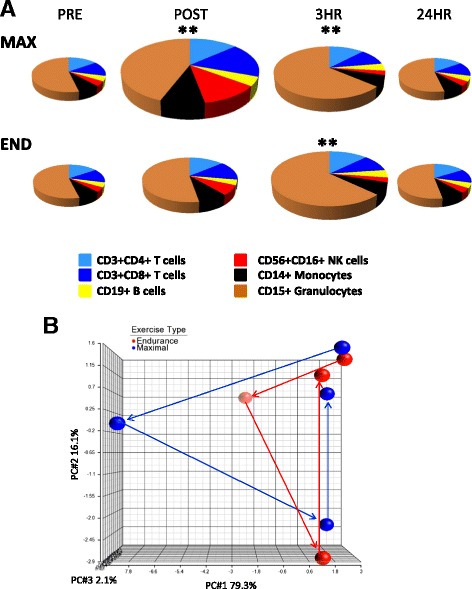
The quantity and composition of peripheral blood leukocytes change in response to different exercises. **a** The mean values of six subsets from 15 subjects were combined to visually recreate the entire peripheral blood compartment and plotted as pie graphs. The pie graphs are sized in relation to the Pre time point prior to maximal exercise. The means were significantly different (*p* < 0.0001 by repeated measures ANOVA). ** = *p* < 0.01 by the Bonferroni’s multiple comparison post-test of all pairs. **b** Principal component plot of the cumulative immune profiles of 15 subjects through each time point for maximal and endurance exercises. Each sphere represents the mean value of 16 immunophenotypes normalized to the baseline sample prior to the maximal exercise

To illustrate the peripheral blood leukocyte system changes in response to exercise, we took the mean values of each major phenotype from the 15 subjects and created pie graphs to reflect the size and composition of the leukocytes at each time point (Fig. 3a). The baseline sample prior to the maximal test was used to set the pie graph at 100% and the subsequent pie graphs were all sized in proportion to the baseline sample. As a whole, maximal exercise resulted in the expansion of cells at immediately post exercise (163%) and remained high at the three hour time point (135%) whereas leukocytes demonstrated slower kinetics with a 122% increase immediately after exercise and peaking to 140% at the three hour time point for the endurance test. After 24 h, the system returned to baseline for both regimens (98% for maximal and 100% for endurance). From another viewpoint, the magnitude and nature of leukocyte populations can be visualized as a whole system. Figure 3b, shows a principal component analysis plot of the composite immune system changes of 15 subjects for each bout of exercise (maximal and endurance) where each sphere represents the immune status (representing 16 phenotypes) at each time point. The movement of the spheres in both magnitude and direction are distinct between the two types of exercise suggesting that a lower intensity, longer work-out doesn’t reflect only a lesser degree of leukocytosis; rather, the composition and magnitude of leukocyte changes appear to be very sensitive to the intensity and duration of the exercise. Taken together, this data reveals that the coordination of mobilized peripheral blood cell populations displays differential kinetics dependent on the nature of the exercise.

### Active versus sedentary individuals

**Fig. 4:**
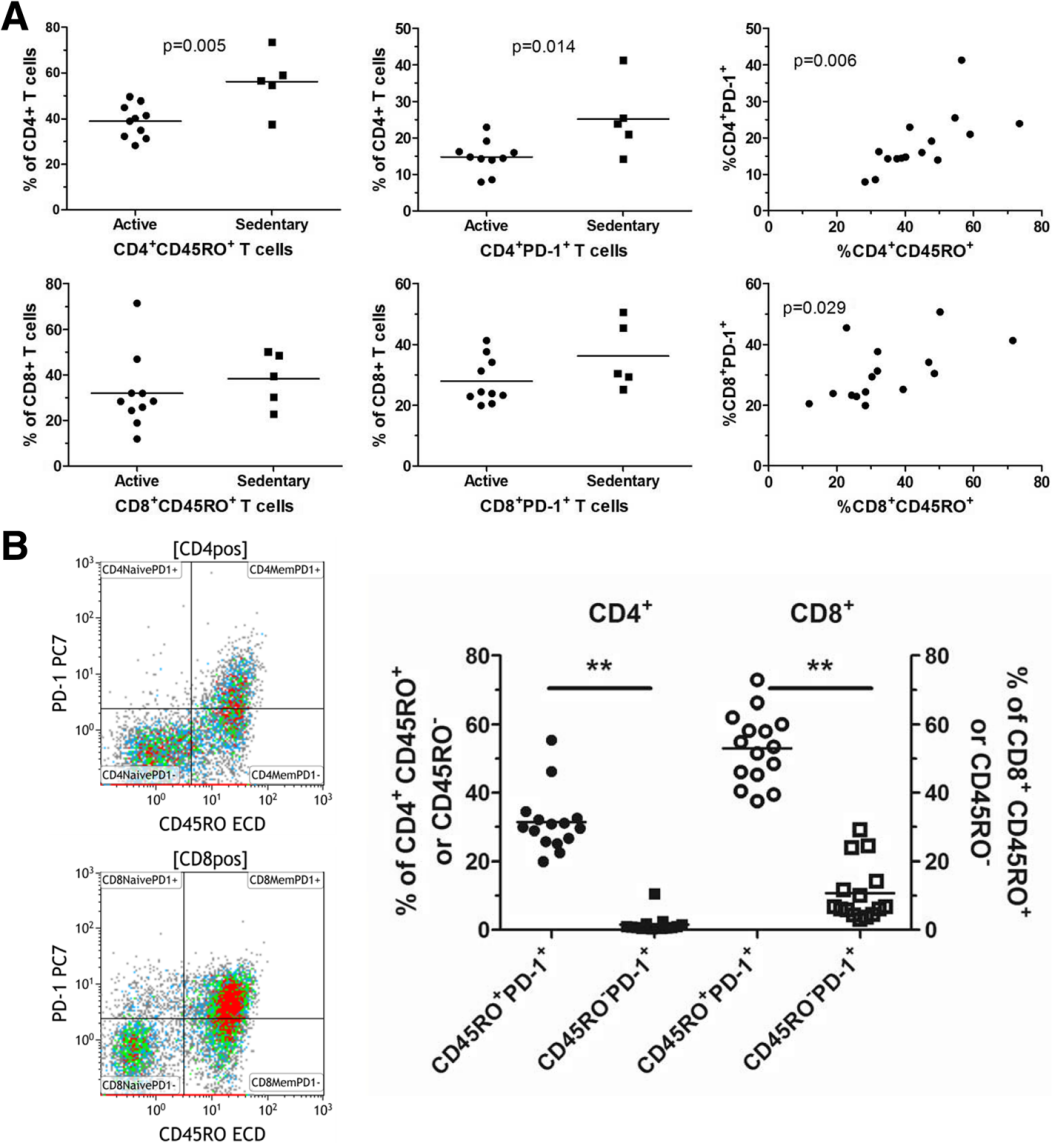
Immunophenotypic differences in sedentary versus active individuals at baseline samples. Immunophenotypes were measured by flow cytometry from baseline samples from 10 active subjects and 5 sedentary subjects. **a** CD45RO^+^ and PD-1^+^ cells were measured as a percent of parent CD4^+^ or CD8^+^ T cells in sedentary and active subjects. All subjects were included on the %CD45RO^+^ versus %PD-1^+^ plot. **b** Representative dot plots showing the distribution of positive and negative T cells for CD45RO and PD-1 (CD4 T cells on top, CD8 T cells on bottom.) The graph shows the percent positive PD-1 cells on memory T cells (CD45RO^+^) or naïve T cells (CD45RO^−^) for both CD4 and CD8. ** = *p* < 0.001 by a paired T test

Exercise causes a certain amount of stress to the body relative to the intensity and duration of the workout. We hypothesized that active individuals may be better suited to handle the stress of a burst of exercise than sedentary individuals. As such, we tested whether exercise-induced leukocytosis was different between active and sedentary individuals. As it was difficult to demarcate the very active versus active individuals, we combined the two groups into one and compared them to the five sedentary individuals. We found no evident differences in the exercise-induced leukocytosis between the groups and each group recovered similarly after exercise (Additional file 4). We also tested if leukocyte compositions differ in baseline samples of peripheral blood of active and sedentary individuals. We looked at sub-population phenotypes as a percent of a parent population of T cells and monocytes. From this analysis, most phenotypes were not different between the two groups at baseline (Additional file 5). However, we found that sedentary individuals had higher frequencies of CD4^+^CD45RO^+^ memory T cells and higher CD4^+^PD-1^+^ T cells than active individuals (Fig. 4a). These same phenotypes were higher on CD8^+^ T cells but were not statistically significant. Interestingly, we found a strong direct correlation of the frequencies of PD-1^+^ and CD45RO^+^CD4^+^ T cells and this relationship held true for CD8^+^ T cells as well. This data led us to look directly at the differences of PD-1 expression on memory (CD45RO^+^) and naïve (CD45RO^-^/CD45RA^+^) cells from the entire cohort of subjects. Representative dot plots of CD45RO and PD-1 on T cells reveal the distinct differences in PD-1 expressed on CD45RO^+^ cells compared to CD45RO^−^ cells (Fig. 4b). For the entire group of subjects, the percentage of PD-1^+^ cells was higher in memory cells than in naïve cells and CD8^+^CD45RO^+^ memory T cells had higher PD-1^+^ cells than CD4^+^CD45RO^+^ memory cells. The data shown here suggest that the intrinsic program for leukocyte mobilization may not be dependent on fitness but that there are phenotypic differences between active and sedentary individuals that reflect the physiologic consequences of inactivity.

### Immunophenotypes related to fitness markers

**Fig. 5:**
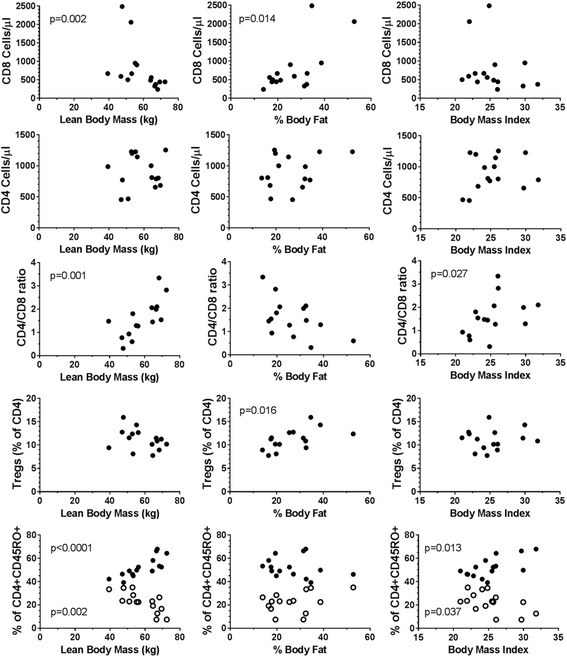
Immunophenotypic associations with fitness parameters. Selected baseline immunophenotypes measured as cell counts or as percentages of a parent population were correlated with three fitness parameters: Lean Body Mass (1st column), % Body Fat (2nd column), and Body Mass Index (BMI, 3rd column). Significant associations are shown with the corresponding p value. The last row of graphs show the percentages of CD4^+^CD45RO^+^CD62L^+^CCR7^+^ central memory T cells (closed circles) and CD4^+^CD45RO^+^CD62L^−^CCR7^−^ effector memory T cells (open circles) from total CD4^+^CD45RO^+^ memory T cells

We used physiological measurements and questionnaires to classify subjects into active and sedentary individuals. One of the sedentary individuals, however, behaved similarly to active individuals during both maximal and endurance tests. As such, there are likely additional intrinsic factors of fitness (or lack of fitness) that influence immune cell populations. Therefore, we assessed phenotypic differences in the cohort divided into very active, active, and sedentary sub-groups. We found no differences when phenotypes were measured from the three different sub-groups. As physical activity may also, but not necessarily, reflect one’s personal fitness, we also investigated whether three fitness markers (Lean Body Mass (LBM), % Body fat, and BMI) were correlated with frequencies of T cell and monocyte phenotypes. CD8^+^ T cells but not CD4^+^ T cells inversely correlated with LBM and % body fat (Fig. 5). Other associations were found with the CD4/CD8 ratio and LBM and BMI, Tregs with % body fat. CD4^+^CD45RO^+^CD62L^+^CCR7^+^ central memory T cells were positively associated with LBM and BMI whereas CD4^+^CD45RO^+^CD62L^−^CCR7^−^ effector memory T cells were inversely associated with LBM and BMI. No associations were seen with monocyte phenotypes (data not shown). The data taken together suggest that in addition to regular exercise, other markers of intrinsic fitness also associate with changes in T cell phenotypes.

## Discussion

Exercise is a mix of intensity, frequency and duration of activity that impacts physical, mental, and emotional health. This study was to determine the influence of intensity/duration and fitness on immune cell populations, both individually and as a whole system. Stronger sympathetic activation occurs with higher intensity exercise, leading to higher heart rates, cardiac outputs, blood pressure, and other measures of physiological stress. However, most people don’t perform high intensity exercise and thus opt for a longer duration and less intense regimen. Thus we compared how a very intense exercise and a less intense, longer duration exercise affected the distribution and magnitude of immune cell populations. Since exercise-induced leukocytosis is a well-established phenomenon, we wanted to assess changes in populations in relation to each other and how the peripheral blood immune system changes as a whole while measuring phenotypes that potentially hinder cancer immunotherapies. Furthermore we sought to determine if we could identify immunological markers representative of physiological inactivity. The subject demographics were limited to relatively young men to control for the effects of age and gender on the immune system.

In response to the maximal test, leukocyte populations peaked immediately after exercise as expected. All leukocyte populations increased immediately after this test. Mononuclear cells returned to baseline levels after 3 h post exercise, however, granulocytes peaked at this time point. Natural killer cells demonstrated the highest degree of induction immediately post exercise. NK cells have been reported to drop below baseline samples after prolonged, intense bouts of exercise like running in a marathon [16]. We did not see a significant decline in NK cells after either regimen but that could likely be explained by the length and intensity of the exercise or perhaps by the timing of the second post exercise sample (3 h). For the endurance test, the response was attenuated compared to the maximal test but the leukocyte populations peaked at 3 h rather than immediately after exercise. As with the maximal test, the induction for granulocytes was different from mononuclear cells by peaking at 3 h. The two different exercise tests thus revealed differential kinetics of mobilization with leukocytes peaking at immediately post exercise for the maximal test and leukocytes peaking at 3 h post exercise for the endurance test. Immune cell populations displayed varying degrees of responsiveness to the intensity, duration, and recovery time of the exercises. Several immune cell populations that had the largest degree of mobilization in our study have also been previously identified as highly responsive to catecholamine-induce mobilization [17]. The infusion of epinephrine into healthy men resulted in the rapid accumulation of CD56^+^CD16^+^ NK cells, CD56^+^CD3^+^ NK-T cells, γδ T cells, CCR7^−^CD45RA^+^CD8^+^ effector T cells, and non-classical CD14^lo^CD16^+^ monocytes. We found CD56^+^CD16^+^ NK cells, CD56^+^CD3^+^ NK-T cells, γδ T cells, and non-classical CD14^lo^CD16^+^ monocytes had the largest degree of mobilization in the maximal regimen (Fig. 2 and data not shown). These pro-inflammatory cells also express high levels of adhesion molecules including CD11a, CD11b, CD62L, and/or CXCR3 that facilitate cellular trafficking and attachment to endothelium [9, 17]. As such, the stronger sympathetic activation leading to more catecholamine release in the maximal regimen may account for the greater induction of the leukocytes than the endurance regimen. It is also possible that with maximal exercise there is a greater rise in cardiac output, greater increase in metabolites such as lactate, more aggressive pH changes as well as potentially greater perfusion of tissues such as bone. Granulocytes, which express CD11b, appear to have a more complex regulation of mobilization and activity where the delayed response may result from the coordinated activities of catecholamines, cytokines, cortisol, and muscle-damage [18, 19]. Immunosuppressive cells were not similarly changed in response to maximal activity. PD-1^+^ CD4^+^ and CD8^+^ T cells and CD14^+^HLA-DR^lo/neg^ monocytes demonstrated small but significant percentage increases, regulatory T cells decreased, and CTLA-4^+^ T cells did not change (Additional file 3). Thus exercise transiently induces largely pro-inflammatory leukocytes primed for rapid immune responses.

We did not observe differences in the induction of cells between active and sedentary individuals. This finding suggests that the mechanisms regulating this response are independent of physiological fitness. Individual differences are likely to contribute to the variation of leukocyte mobilization. For example, one individual that characterized himself as sedentary performed better than what was predicted. Genetics, diet, and other factors including but not limited to CMV exposure [20], may explain this discrepancy. Consequently, the heterogeneity in the responses to exercise in future studies must be taken into consideration and a larger set of subjects is warranted to explore how these factors contribute to the immunological response to exercise.

In both exercise tests, it is remarkable that the 24 h post exercise leukocyte levels were nearly indistinguishable from pre-exercise levels. The mechanisms controlling immune cell homeostasis in humans are both complex and not completely understood. While data is emerging on the efflux of immune cells into the bloodstream during and after exercise, there is very little data regarding how the body controls the process of returning to baseline levels. We have proposed that the immune system acts very similarly to free energy landscapes where each individual immune system exists in a state of stability [13]. Any brief stimulus, such as exercise or an infection, will alter the system and when the stimulus is removed, the system returns to normal. Chronic stimuli, such as autoimmunity, obesity, and even cancer, can fundamentally change homeostatic mechanisms resulting in a new altered baseline. Nevertheless, the processes controlling immune cell homeostasis are highly coordinated and the contributions of many factors are now just beginning to be identified.

While the responses to exercise in active and sedentary subjects were generally similar, we did identify several immunophenotypes that were elevated in sedentary individuals and associated with fitness markers. Sedentary individuals had higher frequencies of CD4^+^CD45RO^+^ memory T cells and higher CD4^+^PD-1^+^ T cells than active individuals. As humans age, the proportion of memory T cells of the total T cell pool greatly increases [21]. In mouse models, exercise training is capable of increasing the naïve to memory T cell ratio in aged animals [22]. PD-1 is expressed on a fraction of T cells and the binding of PD-1 to its ligand PD-L1/CD274/B7-H1 blocks lymphocyte activation [23], induces apoptosis [24] and has been shown to be involved in the regulation of peripheral tolerance [25]. In humans, the precise roles of PD-1 in immune-regulation remain uncertain, although our data demonstrating differential expression of PD-1 naïve and memory T cells would suggest a likely role in regulating the homeostasis of these cell populations. Interestingly, we found elevated PD-1^+^CD4^+^ T cells in sedentary individuals but found no associations with PD-1 and fitness parameters related to obesity (i.e. % body fat and BMI). Therefore, although the consequences of higher frequencies of these cells may lead to sub-optimal immune responses, further research is required to delineate how a sedentary life-style negatively impacts immune responses in humans. Subjects with lower LBM and higher body fat had higher CD8^+^ T cell counts. CD8^+^ cells have previously been implicated in obesity-mediated inflammation [26]. We also found that immunosuppressive Tregs (CD4^+^CD25^+^CD127^lo^) were higher in subjects with high body fat. These immunophenotypes were found to be associated with fitness parameters related to obesity even though only one of the subjects in the study had a BMI above 30. Data from both animal models and human studies have revealed several immune cell populations altered by obesity. Nieman et al. reported that neutrophils, monocytes and T cells were elevated in obese subjects [27]. Other tissue resident immune cells have since been implicated in obesity-induced inflammation including macrophages, B cells, NK cells, and regulatory T cells [28, 29]. Thus, the effects of excess body fat may disrupt the intricate balance between positive and negative immune regulators. Unlike the beneficial pro-inflammatory response to moderate exercise, the pro-inflammatory state caused by visceral adipose tissue leads to detrimental immune responses resulting in increased susceptibility to type II diabetes, cancer, and/or infections. Although our data identified distinct immunophenotypes related to sedentary lifestyles in mainly non-obese subjects, the connection between physical inactivity and obesity is strong. Therefore, more data is needed to further delineate immune dysfunction caused by lack of physical activity compared to dysfunction caused by metabolomics.

There is now a substantial amount of published studies that provide evidence that physical activity improves the overall health of cancer patients and may prolong survival. As the evidence for the positive effects of long term moderate exercise continues to be determined, acute exercise regimens could be employed to potentially improve both traditional cancer therapy and immunotherapeutic approaches. In rodent models, wheel running reduced tumor formation and growth through the induction of pro-inflammatory pathways [30]. Specifically, epinephrine-sensitive NK cells were recruited to tumors through an IL-6 mediated mechanism. Regular exercise also appears to improve T cell responses to antigens by increasing cytokine production and cellular proliferation [31]. In the human setting of autologous peripheral hematopoietic blood stem cell transplantation (APHSCT), Porrata et al. demonstrated a significant improvement in clinical outcomes for those patients infused with an absolute lymphocyte count (ALC) above 0.5 × 10^9^ kg and additionally that higher infused NK cell counts were associated with improved clinical outcomes [32]. They noted that additional methodologies are needed to increase the ALC dose. Since acute exercise mobilizes lymphocytes, and NK cells to a much larger degree, a brief exercise regimen may be a novel way to increase NK cell and lymphocyte counts to obtain the appropriate amount of cells needed to extend survival in transplant patients. In addition to APHSCT, acute exercise may provide a unique way to increase the number of cells collected for other cellular based immunotherapies like adoptive T cell therapies [33] and monocytes for dendritic cell therapies [34].

## Conclusions

The immune system shows remarkable plasticity in response to exercise. The data presented here reveal the differential coordination of mobilized cells into peripheral blood. This acute coordination did not appear to be dependent on the activity level of the subjects. However, the activity level did appear to affect distinct cellular immunophenotypes in baseline samples. In light of the fact that a fourth of cancer cases are attributed to obesity and lack of physical activity [4], our data provide mechanistic clues to this phenomenon through immune dysfunction mediated by an altered balance of positive and negative immune regulators like CD8^+^ T cells, memory T cells, PD-1^+^ T cells, and regulatory T cells. Ultimately, exercise may be used as a tool to optimize the immune system for patients receiving cancer immunotherapy and other patients exhibiting abnormal immunity caused by obesity, infections, or other diseases.

## Additional files

Additional file 1: Protocols for T cell and Monocyte phenotypes. Information regarding additional antibodies used for flow cytometry and instrument settings. (PDF 180 kb)

Additional file 2: Activity data for participants from the Body Media device and questionnaires. (PDF 30 kb)

Additional file 3: Immunophenotype values in pre and post-maximal exercise. (PDF 11 kb)

Additional file 4: The mobilization of leukocyte subsets does not appear to be significantly different in active subjects and sedentary subjects. (PDF 299 kb)

Additional file 5: Immunophenotype values in active and sedentary individuals. (PDF 17 kb)

## Abbreviations

BP: Blood pressure
DEXA: Dual-energy X-ray absorptiometry
FVC: Flow-volume curves
HR: Heart rate
LBM: Lean body mass
MET: Measure of energy expenditure based on oxygen consumption
PETCO_2_: Partial pressure of end-tidal carbon dioxide
RER: Respiratory exchange ratio
RPE: Rate of perceived exertion, Tregs, regulatory T cells
VE: Minute ventilation
