# Supplementary material for: A systems biology approach to investigating the influence of exercise and fitness on the composition of leukocytes in peripheral blood
Source: J Immunother Cancer. 2017 Apr 18;5:30. doi: 10.1186/s40425-017-0231-8 (PMC5394617; doi:10.1186/s40425-017-0231-8)
Supplement: Supplementary file 1 — Protocols for T cell and Monocyte phenotypes. Information regarding additional antibodies used for flow cytometry and instrument settings. (PDF 180 kb) [file 40425_2017_231_MOESM1_ESM.pdf]

A

Protocol	FITC	PE	ECD	PC5.5	PC7	APC	APC-700	APC-750	Pac Blu	KrO
T Cell-2	CD154*	CD152*	CD45RO	CD56	CD279	CD3	CD8	CD28	CD4	CD45
Monocytes	CD40*	CD142*	CD14	CD33	CD274	CD86*		CD16	HLA-DR	CD45

B

	FS	SS	FL1	FL2	FL3	FL4	FL5	FL6	FL7	FL8	FL9	FL10
Voltage	205	527	452	421	459	565	624	570	515	498	386	358
Gain	5.0	7.5	1.0	1.0	1.0	1.0	1.0	1.0	1.0	1.0	1.0	1.0
	**FS**	**SS**	**FL1**	**FL2**	**FL3**	**FL4**	**FL5**	**FL6**	**FL7**	**FL8**	**FL9**	**FL10**
Discriminator	90	OFF	OFF	OFF	OFF	OFF	OFF	OFF	OFF	OFF	OFF	OFF
	**FL1**	**FL2**	**FL3**	**FL4**	**FL5**	**FL6**	**FL7**	**FL8**	**FL9**	**FL10**		
FL1		1.4	0.2	0.1	0.1	0	0	0	0	1.4		
FL2	14.9		7.2	0.7	0.7	0	0	0	0	0		
FL3	7.9	44.5		0.1	0.6	0	0	0	0	0		
FL4	1	9.5	55.9		0	0.6	0.5	0	0	0		
FL5	0	1	10.5	33.6		0.5	0.8	3.6	0	0		
FL6	0	0	0.4	1.1	0		12.1	15.2	0	0		
FL7	0	0	0.1	15.7	0.4	19.7		5.9	0	0		
FL8	0	0	0	6	6.3	5.7	30.3		0	0		
FL9	0	0	0	0	0	0	0	0		2.3		
FL10	1	1.5	0	0	0	0	0	0	5.9			
**Lasers**	**Status**	**Shutter**	**Power**	**Current**								
Blue	On	Closed	22 mW	0.43 A								
Red	On	Closed	25 mW	0.07 A								
Violet	On	Closed	40.1 mW	0.07 A								

C

	FS	SS	FL1	FL2	FL3	FL4	FL5	FL6	FL7	FL8	FL9	FL10
Voltage	208	654	432	422	447	581	629	593	516	492	354	326
Gain	5.0	7.5	1.0	1.0	1.0	1.0	1.0	1.0	1.0	1.0	1.0	1.0
	**FS**	**SS**	**FL1**	**FL2**	**FL3**	**FL4**	**FL5**	**FL6**	**FL7**	**FL8**	**FL9**	**FL10**
Discriminator	100	OFF	OFF	OFF	OFF	OFF	OFF	OFF	OFF	OFF	OFF	OFF
	**FL1**	**FL2**	**FL3**	**FL4**	**FL5**	**FL6**	**FL7**	**FL8**	**FL9**	**FL10**		
FL1		1.4	0.1	0.2	0.1	2	0	0	0	1.4		
FL2	14		8.2	0.5	1.3	0.7	0	0	0	0		
FL3	3.2	44.5		0	0.6	0	0	0	0	0		
FL4	1	9.5	3.8		0.2	0.6	0.5	0	0	0		
FL5	0	1	1	26.2		1.4	0	1.9	0	0		
FL6	22.2	0	0.4	2.3	1.6		6.9	23.3	0	0		
FL7	0	0	0	2.3	0.4	14.7		9	0	0		
FL8	0	0	0	3.2	10.8	2	23.1		0	0		
FL9	0	0	0	0	0	0	0	0		2.3		
FL10	1	1.5	0	0	0	0	0	0	5.9			
**Lasers**	**Status**	**Shutter**	**Power**	**Current**								
Blue	On	Closed	22 mW	0.43 A								
Red	On	Closed	24.9 mW	0.07 A								
Violet	On	Closed	40 mW	0.07 A								

Additional File 1. Protocols for T cell and Monocyte phenotypes. **A.** List of antibodies. All antibodies were purchased from Beckman Coulter unless otherwise designated. *= antibodies purchased from BD Biosciences. **B.** Beckman Coulter Gallios instrument settings for the T-cell-2 protocol. **C.** Beckman Coulter Gallios instrument settings for the Monocytes protocol.
