# Supplementary material for: A systems biology approach to investigating the influence of exercise and fitness on the composition of leukocytes in peripheral blood
Source: J Immunother Cancer. 2017 Apr 18;5:30. doi: 10.1186/s40425-017-0231-8 (PMC5394617; doi:10.1186/s40425-017-0231-8)
Supplement: Supplementary file 2 — Activity data for participants from the Body Media device and questionnaires. (PDF 30 kb) [file 40425_2017_231_MOESM2_ESM.pdf]

	Very active	Active	Sedentary
Total EE (avg kj)	13529±2272	12690±3560	11248±2420
Active EE >3.0MET (avg kj)	4059±1193	3750±2758	2093±979
Avg # of steps	10844±3341	8964±3958	7815±2412
Length of regular exercise (years)	12.3±9.5	7.8±5.8	N/A
Total aerobic exercise (mins per week)	336±176	648±498	N/A
Total run/cycle (mins per week)	270±157	291±232	N/A
Total resistance exercise (mins per week)	165±114	156±88	N/A

Additional File 2.. Activity data for participants from the Body Media device and questionnaires.
