# Supplementary material for: A systems biology approach to investigating the influence of exercise and fitness on the composition of leukocytes in peripheral blood
Source: J Immunother Cancer. 2017 Apr 18;5:30. doi: 10.1186/s40425-017-0231-8 (PMC5394617; doi:10.1186/s40425-017-0231-8)
Supplement: Supplementary file 3 — Immunophenotype values in pre and post-maximal exercise. (PDF 11 kb) [file 40425_2017_231_MOESM3_ESM.pdf]

Additional File 3. Immunophenotype values in pre and post-maximal exercise.

Phenotype	Pre (Mean + SD)	Post (Mean + SD)	P value
T cell Phenotypes			
CD4+CD25+CD127lo Regulatory T cells (% of CD4)	11.26 ±2.31	10.18 ±2.24	0.006
CD4+CD45RA+ Naïve T cells (% of CD4)	44.78 ±12.66	41.68 ± 12.45	0.006
CD4+CD45RO+ Memory T cells (% of CD4)	45.67 ±12.19	49.24 ±12.42	0.002
CD4+CD45RO+CD62L+CCR7+ Central Memory (% of CD4+CD45RO+)	51.20 ±8.25	43.36 ±9.673	0.004
CD4+CD45RO+CD62L-CCR7- Effector Memory (% of CD4+CD45RO+)	23.42 ±7.98	30.50 ±11.41	0.003
CD4+CD62L+CD27+ (% of CD4)	89.46 ±11.65	80.64 ±20.72	0.005
CD4+CD25+ (% of CD4)	7.99 ±3.71	8.17 ±3.45	0.79
CTLA-4+CD28- (% of CD4)	0.16 ±0.19	0.21 ±0.25	0.339
CTLA-4+CD28+ (% of CD4)	2.17 ±3.05	1.90 ±2.11	0.84
CTLA-4-CD28+ (% of CD4)	92.91 ±8.06	87.87 ±15.61	0.0001
CD4+PD-1+ (% of CD4)	18.28 ±8.16	21.43 ±10.72	0.0001
CD154(CD40L)+ (% of CD4)	2.76 ±3.22	2.51 ±2.47	0.45
CD8+CD45RA+ Naïve T cells (% of CD8)	64.65 ±15.39	63.37 ±16.57	0.41
CD8+CD45RO+ Memory T cells (% of CD8)	34.79 ±15.36	36.02 ±16.45	0.40
CD8+CD45RO+CD62L+CCR7+ Central Memory (% of CD8+CD45RO+)	9.02 ±5.36	6.04 ±3.51	0.001
CD8+CD45RO+CD62L-CCR7- Effector Memory (% of CD8+CD45RO+)	74.63 ±12.24	79.26 ±10.97	0.003
CD8+CD62L+CD27+ (% of CD8)	43.75 ±17.77	29.18 ±14.14	<0.0001
CD8+CD25+ (% of CD8)	1.40 ±0.77	1.11 ±0.50	0.09
CD8+CD45RA+CD27+CD62L+CCR7+	24.83	14.71	0.0003

Stem Cell Memory (% of CD8+CD45RA+CD27+)	±13.30	±9.24	
CTLA-4+CD28- (% of CD8)	0.74 ±2.0	0.42 ±0.50	0.19
CTLA-4+CD28+ (% of CD8)	1.59 ±3.02	0.88 ±0.66	0.32
CTLA-4-CD28+ (% of CD8)	63.35 ±14.44	54.10 ±14.97	<0.0001
CD8+PD-1+ (% of CD8)	30.70 ±9.45	36.03 ±11.51	0.0001
CD154(CD40L)+ (% of CD8)	1.77 ±1.45	1.41 ±0.92	0.11
Monocyte Phenotypes			
CD33+ (% of Mononuclear cells)	17.96 ±7.24	15.67 ±6.09	0.048
CD14+ (% of CD33+ cells)	80.73 ±5.96	81.32 ±4.54	0.89
CD33+CD14-HLA-DR- Immature myeloid derived suppressor cells (% of CD33+ cells)	11.16 ±4.34	9.61 ±3.32	0.14
CD14+HLA-DRlo/neg monocytes (% of CD14+)	12.58 ±11.02	13.72 ±11.22	0.035
HLA-DR Geometric Mean* (on CD14+HLA-DRlo/neg monocytes)	3.09 ±1.06	3.13 ±1.11	0.94
CD86+ monocytes (% of CD14+)	98.75 ±0.88	97.85 ±2.73	0.0012
CD40+ monocytes (% of CD14+)	5.59 ±3.59	6.86 ±4.91	0.06
B7-H1 (PD-1 ligand)+ monocytes (% of CD14+)	3.12 ±2.32	2.27 ±1.51	0.048
CD142 (Tissue Factor)+ (% of CD14+)	4.72 ±4.63	3.98 ±3.44	0.27
Classical Monocytes (CD14+CD16-) (% of CD14+)	78.70 ±7.31	74.88 ± 5.44	0.005
HLA-DR Geometric Mean (on Classical Monocytes (CD14+CD16-)	2.69 ± 0.90	2.66 ±0.98	0.30
Intermediate Monocytes (CD14+CD16+) (% of CD14+)	5.37 ± 1.84	6.31 ± 2.07	0.06
HLA-DR Geometric Mean (on Intermediate Monocytes (CD14+CD16+)	13.98 ±6.68	14.25 ±7.59	0.89
Non-classical Monocytes (CD14loCD16+) (% of CD14+)	6.77 ±3.43	9.26 ±3.45	<0.0001
HLA-DR Geometric Mean (on Non-classical Monocytes (CD14loCD16+)	6.66 ±3.19	6.30 ±2.96	0.33

* Geometric means are presented in mean fluorescence intensity units.
