# Supplementary material for: A systems biology approach to investigating the influence of exercise and fitness on the composition of leukocytes in peripheral blood
Source: J Immunother Cancer. 2017 Apr 18;5:30. doi: 10.1186/s40425-017-0231-8 (PMC5394617; doi:10.1186/s40425-017-0231-8)
Supplement: Supplementary file 4 — The mobilization of leukocyte subsets does not appear to be significantly different in active subjects and sedentary subjects. (PDF 299 kb) [file 40425_2017_231_MOESM4_ESM.pdf]

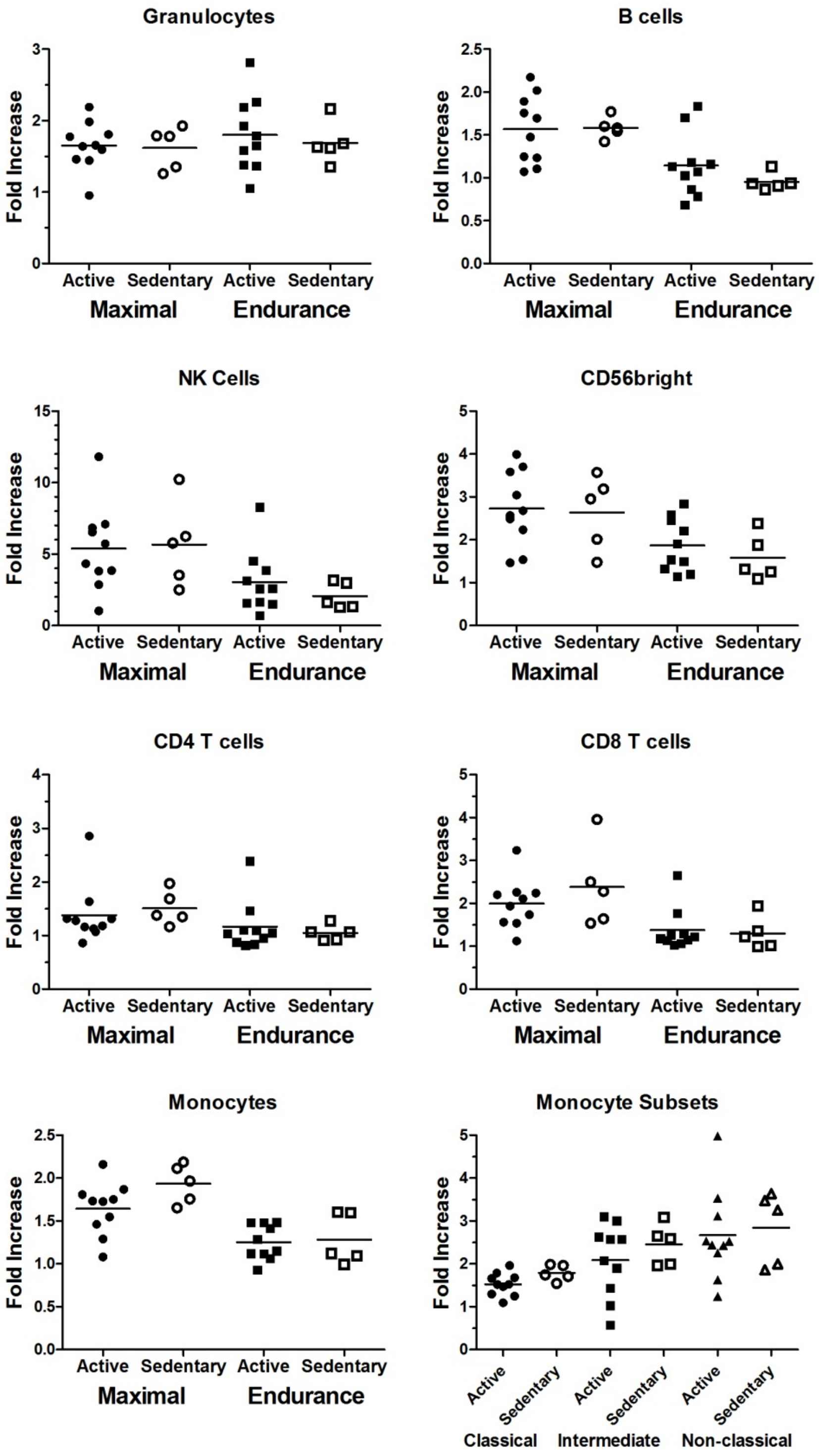

Additional File 4. The mobilization of leukocyte subsets does not appear to be significantly different in active subjects and sedentary subjects. For each subset, the increase from pre samples to post samples is shown for both maximal and endurance exercises except for Granulocytes (Pre vs 3HR) and for the monocyte subsets in which only the maximal Pre samples are shown.
