# Supplementary material for: A systems biology approach to investigating the influence of exercise and fitness on the composition of leukocytes in peripheral blood
Source: J Immunother Cancer. 2017 Apr 18;5:30. doi: 10.1186/s40425-017-0231-8 (PMC5394617; doi:10.1186/s40425-017-0231-8)
Supplement: Supplementary file 5 — Immunophenotype values in active and sedentary individuals. (PDF 17 kb) [file 40425_2017_231_MOESM5_ESM.pdf]

Additional File 5. Immunophenotype values in active and sedentary individuals.

Phenotype	Active (n=10) Mean and SD	Sedentary (n=5) Mean and SD
Granulocytes (cells/µl) (FSCbySSC)	2897.6 ± 1183.1	3318.4 ± 680.2
Neutrophils (CD15+CD16+) (cells/µl)	2753.9 ± 1190.9	3141.1 ± 584.2
Eosinophils (CD15+CD16-) (cells/µl)	141.7 ± 87.9	173.8 ± 97.8
Monocytes (CD14+) (cells/µl)	447.6 ± 151.7	516.3 ± 114.9
Classical Monocytes (CD14+CD16-) (cells/µl)	376.3 ± 129.7	419.8 ± 94.2
Intermediate Monocytes (CD14+CD16+) (cells/µl)	22.3 ± 9.9	36.4 ± 9.1
Non-classical Monocytes (CD14loCD16+) (cells/µl)	38.5 ± 14.8	50.7 ± 23.9
B cells (CD19+) (cells/µl)	241.3 ± 99.9	344.1 ± 111.6
Natural Killer cells (CD56+CD16+) (cells/µl)	277.8 ± 195.6	209.8 ± 72.6
Natural Killer cells (CD56++CD16-) (cells/µl)	24.8 ± 14.8	20.3 ± 8.2
T cells (CD3+) (cells/µl)	1791.4 ± 976.6	1911.9 ± 924.2
T helper cells (CD3+CD4+) (cells/µl)	923.5 ± 280.1	848.8 ± 281.4
T cytotoxic cells (CD3+CD8+) (cells/µl)	672.8 ± 520.4	996.1 ± 859.6
T cells (CD3+CD4+CD8+) (cells/µl)	21.2 ± 28.7	14.3 ± 5.6
T cells (CD3+CD4-CD8-) (cells/µl)	173.8 ± 295.0	52.7 ± 14.4
δΔ T cells (CD3+δΔTCR+)	187.5 ± 336.8	35.9 ± 14.2
CD4/CD8 ratio	1.8 ± 0.8	1.2 ± 0.7
T cell Phenotypes		
CD4+CD25+CD127lo Regulatory T cells (% of CD4)	10.6 ± 1.9	12.4 ± 2.7
CD4+CD45RA+ Naïve T cells (% of CD4)	50.0 ± 10.4	37.3 ± 13.9
CD4+CD45RO+CD62L+CCR7+	53.3	49.6

Central Memory (% of CD4+CD45RO+)	± 7.4	± 11.2
CD4+CD45RO+CD62L-CCR7- Effector Memory (% of CD4+CD45RO+)	20.9 ± 8.7	25.2 ± 9.0
CD4+CD62L+CD27+ (% of CD4)	90.4 ± 13.5	89.5 ± 7.1
CD4+CD25+ (% of CD4)	7.5 ± 3.8	9.5 ± 3.3
CTLA-4+CD28- (% of CD4)	0.1 ± 0.1	0.1 ± 0.1
CTLA-4+CD28+ (% of CD4)	1.1 ± 0.7	2.1 ± 1.7
CTLA-4-CD28+ (% of CD4)	94.4 ± 8.5	93.0 ± 5.5
CD154(CD40L)+ (% of CD4)	2.6 ± 2.4	1.0 ± 0.3
CD8+CD45RA+ Naïve T cells (% of CD8)	67.6 ± 16.7	60.9 ± 11.4
CD8+CD45RO+ Memory T cells (% of CD8)	32.0 ± 16.6	38.3 ± 11.8
CD8+CD45RO+CD62L+CCR7+ Central Memory (% of CD8+CD45RO+)	11.6 ± 5.7	5.8 ±3.5
CD8+CD45RO+CD62L-CCR7- Effector Memory (% of CD8+CD45RO+)	69.1 ± 12.7	81.3 ± 10.7
CD8+CD62L+CD27+ (% of CD8)	48.6 ± 13.0	33.1 ± 21.2
CD8+CD25+ (% of CD8)	1.7 ± 0.9	1.0 ± 0.4
CD8+CD45RA+CD27+CD62L+CCR7+ Stem Cell Memory (% of CD8+CD45RA+CD27+)	29.2 ± 11.1	16.5 ± 12.8
CTLA-4+CD28- (% of CD8)	0.2 ± 0.2	0.2 ± 0.2
CTLA-4+CD28+ (% of CD8)	0.8 ± 0.4	0.9 ± 0.5
CTLA-4-CD28+ (% of CD8)	65.8 ± 11.7	63.4 ± 17.8
CD154(CD40L)+ (% of CD8)	2.3 ± 1.7	1.0 ± 0.3
Monocyte Phenotypes		
CD33+ (% of Mononuclear cells)	17.9 ± 6.6	18.0 ± 9.2
CD14+ (% of CD33+ cells)	81.7	78.8

	± 4.5	± 8.5
CD33+CD14-HLA-DR- Immature myeloid derived suppressor cells (% of CD33+ cells)	9.6 ± 2.7	14.4 ± 5.5
CD14+HLA-DRlo/neg monocytes (% of CD14+)	11.8 ± 9.1	14.1 ± 15.2
HLA-DR Geometric Mean* (on CD14+HLA-DRlo/neg monocytes)	3.2 ± 1.1	2.9 ± 1.1
CD86+ monocytes (% of CD14+)	98.7 ± 1.0	98.8 ± 0.8
CD40+ monocytes (% of CD14+)	5.7 ± 4.2	5.3 ± 2.2
B7-H1 (PD-1 ligand)+ monocytes (% of CD14+)	3.2 ± 2.6	3.1 ± 2.0
CD142 (Tissue Factor)+ (% of CD14+)	4.9 ± 5.3	4.3 ± 3.5
Classical Monocytes (CD14+CD16-) (% of CD14+)	78.4 ± 7.2	79.4 ± 8.4
HLA-DR Geometric Mean (on Classical Monocytes (CD14+CD16-)	2.8 ± 0.9	2.5 ± 0.9
Intermediate Monocytes (CD14+CD16+) (% of CD14+)	5.1 ± 2.0	5.8 ± 1.4
HLA-DR Geometric Mean (on Intermediate Monocytes (CD14+CD16+)	13.9 ± 6.8	14.2 ± 7.3
Non-classical Monocytes (CD14loCD16+) (% of CD14+)	6.8 ± 3.5	6.7 ± 3.8
HLA-DR Geometric Mean (on Non-classical Monocytes (CD14loCD16+)	6.5 ± 3.2	6.7 ± 3.8

* Geometric means are presented in mean fluorescence intensity units.
